# Asymmetric Proteome Equalization of the Skeletal Muscle Proteome Using a Combinatorial Hexapeptide Library

**DOI:** 10.1371/journal.pone.0028902

**Published:** 2011-12-19

**Authors:** Jenny Rivers, Chris Hughes, Thérèse McKenna, Yvonne Woolerton, Johannes P. C. Vissers, James I. Langridge, Robert J. Beynon

**Affiliations:** 1 Protein Function Group, Institute of Integrative Biology, University of Liverpool, Liverpool, United Kingdom; 2 Waters Corporation MS Technologies Centre, Atlas Park, Wythenshawe, Manchester, United Kingdom; University of South Florida College of Medicine, United States of America

## Abstract

Immobilized combinatorial peptide libraries have been advocated as a strategy for equalization of the dynamic range of a typical proteome. The technology has been applied predominantly to blood plasma and other biological fluids such as urine, but has not been used extensively to address the issue of dynamic range in tissue samples. Here, we have applied the combinatorial library approach to the equalization of a tissue where there is also a dramatic asymmetry in the range of abundances of proteins; namely, the soluble fraction of skeletal muscle. We have applied QconCAT and label-free methodology to the quantification of the proteins that bind to the beads as the loading is progressively increased. Although some equalization is achieved, and the most abundant proteins no longer dominate the proteome analysis, at high protein loadings a new asymmetry of protein expression is reached, consistent with the formation of complex assembles of heat shock proteins, cytoskeletal elements and other proteins on the beads. Loading at different ionic strength values leads to capture of different subpopulations of proteins, but does not completely eliminate the bias in protein accumulation. These assemblies may impair the broader utility of combinatorial library approaches to the equalization of tissue proteomes. However, the asymmetry in equalization is manifest at either low and high ionic strength values but manipulation of the solvent conditions may extend the capacity of the method.

## Introduction

Although the capability of mass spectrometry based proteomics has been greatly enhanced, the challenge of dynamic range is not fully solved. Individual proteins in a proteome are expressed at dramatically different levels, and the low abundance, or ‘deep’ proteome remains elusive. In blood plasma, the dynamic range of protein abundance may span as much as 12 orders of magnitude [1], [2] and even in simple cellular systems such as yeast cytosol, expression may range over four orders of magnitude [3], [4]. The most common approach to global proteome analysis is based on LC-MS/MS of tryptic peptides. The dynamic range of a typical LC-MS/MS analysis is such that it is not feasible to accommodate the range of protein expression levels in a typical tissue preparation. In particular, the low abundance proteins are difficult to analyze because of limited instrument sensitivity and ‘crowding’ of the analyte stream by high abundance peptides that trigger data dependent acquisition and which may cause ion suppression. Data independent data acquisition can ease this problem, but not completely.

Prefractionation and enrichment of subclasses of proteins such as those decorated with specific post-translational modifications can give selective enrichment [5], but can introduce the possibility of higher variance due to sample preparation. More commonly and generically, two strategies of selective proteome manipulation have been employed. First, depletion strategies are subtractive methods that use affinity methods, usually based on antigen∶antibody interaction for selective removal of abundant proteins from a sample [6], [7]. Almost without exception, this approach has been applied to human blood plasma [8]–[10] and has the goal of removal of major protein species that contribute the most intense ions in an LC-MS/MS analysis. Whilst such approaches can deplete abundant proteins (provided appropriate high specificity antibodies are available) they are not capable of enriching trace proteins, and under certain conditions can be non-specific in removing selected high abundance proteins [11].

The second common approach to reduction of the dynamic range of a complex mixture of proteins is that of sample equalization [12]–[20]. This method uses combinatorial peptide libraries to generate a complex set of bead-immobilized ligands, each of which is able to bind, with variable affinity, a small subset of proteins in the analyte. A library of linear hexapeptides based on 20 naturally occurring amino acids has the potential to create a library of 64 million different ligands. Because each ligand is present at low abundance, the outcome is that high abundance proteins are bound to the beads at a finite capacity, and provided that adequate starting material is applied to the beads, low abundance proteins will eventually bind a similar number of beads, the outcome of which is to equalize the protein mixture attached to the beads.

Proteome equalization has predominantly been applied to analysis of human blood plasma, but lesserly to urine [12], [13] and serum [15], [21] as well as non-blood samples including cell and tissue lysates [22]–[27]. However, the emphasis on secreted biofluids, and in particular blood plasma has tended to direct focus to the specific challenges associated with this material and in particular, the reduction in the signal for serum albumin, antibodies and other major plasma proteins. In tissues, protein dynamic range is often not as exaggerated as in plasma. However, one tissue in particular, skeletal muscle, exhibits an asymmetry in the dynamic range of protein expression that is probably as extreme as blood plasma. This is due to two dominant fractions – the contractile apparatus and the sarcoplasmic fraction. The contractile apparatus (predominantly actin and myosin) is readily removed from skeletal muscle preparations as it is insoluble under low ionic strength conditions [28], [29], but the residual component - the soluble protein fraction - also betrays a remarkable degree of specialization. One-dimensional or two-dimensional gel electrophoresis emphasizes the high level expression of a few tens of proteins, largely comprising the glycolytic enzymes responsible for fuelling muscle contraction – see for example [30]–[35]. In fact, the soluble fraction from skeletal muscle might present as much of a challenge in terms of proteome dynamic range as does blood plasma.

It is thought that combinatorial equalization might not be compatible with absolute quantification, because of the finite capacity of the library for each protein, and because experiments are designed to suppress high abundance proteins, allowing measurement of low abundance components. Thus, high abundance proteins saturate the beads, and cannot be quantified. However, low abundance proteins might be accumulated on the library in sufficiently a linear fashion to permit sub-saturating quantification. That is only possible if the binding of the analyte is unaffected by the total protein exposure to the beads or the time of exposure. In this study, we applied the combinatorial equalization library to the *soluble* proteins of chicken skeletal muscle, a tissue that we have studied previously [31], [35], [36]. In particular, we set out to explore the outcome of an exaggerated over-saturation of the library to input protein pool. By the use of label-free [37]–[42] and QconCAT-based [35], [43], [44] analyses, we have uncovered behaviors, not previously seen in studies of extracellular fluids such as blood plasma, that may complicate the equalization of some types of cellular proteins.

## Methods

### Sample preparation

To isolate the soluble fraction of chicken skeletal muscle, supermarket purchased chicken breast (2 g) tissue was homogenized in 18 mL 20 mM sodium phosphate buffer, pH 7.0 containing protease inhibitors (Complete Protease Inhibitors, Roche, Lewes, UK). This was centrifuged at 15,000×*g* for 45 min at 4°C. The supernatant fraction, containing soluble protein, was then removed. The total protein concentration of the final preparation was measured using a Coomassie Plus Protein Assay (Pierce, Northumberland, UK).

### Equalization of proteins using Prospectrum-2 beads

In preliminary sets of experiments, Prospectrum-2 library beads (a similar library is available as ProteoMiner™ Protein Enrichment Kits, Biorad, Hemel Hempstead, UK) were washed in 20 mg batches in 1 mL 50% (v/v) MeOH and mixed gently for 10 min. The beads were allowed to settle and the supernatant was removed and discarded. Methanol (50% (v/v)) was added to cover the surface of the beads that were then allowed to swell overnight at 4°C. Once swollen, 20 mg beads constituted 100 µL settled bed volume and this was transferred to a 1.5 mL Eppendorf tube. Beads were washed in 1 mL ddH_2_O for 30 min prior to equilibration in 20 mM sodium phosphate buffer pH 7.0 for 30 min. After each wash, beads were allowed to settle for 5 min after which the supernatant was removed. Approximately 1 mL of protein mixtures containing 20 mg, 50 mg and 100 mg soluble protein from chicken skeletal muscle was added to the library in parallel experiments and mixed for 2 h. Unbound protein was collected as the supernatant fraction after the beads had been allowed to settle. The beads were subsequently washed five times in 20 mM phosphate buffer and supernatant fractions were removed and collected.

In a second series, designed to test the effect of extreme loading of beads, Prospectrum-2 beads, washed as described previously, were exposed to increasing amounts of soluble proteins from chicken skeletal muscle; chicken pectoralis (30 g) tissue was homogenized in 30 mL 20 mM sodium phosphate buffer, pH 7.0 containing protease inhibitors (Complete Protease Inhibitors, Roche, Lewes, UK). This was centrifuged at 15,000×g for 45 minutes at 4°C. The supernatant fraction, containing soluble protein was then removed. The total protein concentration of the final preparation was measured using a Coomassie Plus Protein Assay (Pierce, Northumberland, UK).

Increasing amounts of protein (20, 50, 100, 250, 500 and 1000 mg) were incubated with the library (in six parallel experiments) for 2 h. For increased protein loading (500 and 1000 mg), beads were incubated for an hour with less material (50% for 500 mg, 25% for 1000 mg), the unbound fraction was removed and the library was incubated with the same volume again of soluble protein; this was repeated four times for total exposure to the beads of 500 mg or 1000 mg protein. The beads were subsequently washed five times in 20 mM phosphate buffer and supernatant fractions were removed and collected. Beads were re-suspended in 200 µL phosphate buffer prior to analysis by 1D SDS-PAGE and mass spectrometry. Starting material, unbound protein, wash fractions and beads containing bound protein were analyzed using 1D SDS-PAGE. Approximately 10 µg protein from each fraction was loaded onto a 12.5% (w/v) acrylamide gel which was run at 200 V for 45 min.

In a final series, designed to test the effect on ionic strength on loading extent and complexity, increasing amounts of proteins (5, 10 and 25 mg) were incubated with 10 µL beads in one of two buffers; a low ionic strength buffer of 20 mM sodium phosphate buffer, pH 7.5 (ionic strength 50 mM) or a high ionic strength buffer of 20 mM sodium phosphate buffer, pH 7.5 containing 150 mM NaCl (ionic strength 200 mM). After incubation for 120 min at room temperature, the beads were washed five times in the binding buffer and the protein that remained attached to the beads was digested as described below for label-free quantification.

### Label free quantification

For label-free quantification, samples (500 ng, 1 µL) from the protein loading study were resolved using a nano-ACQUITY system (Waters Corporation, Milford, MA, USA), equipped with a Symmetry C18 5 µm, 5 mm×300 µm precolumn and an Atlantis C18 3 µm, 15 cm×75 µm analytical RP column (Waters Corporation). The samples, 1 µL partial loop injection, were loaded with 0.1% formic acid solution at 4 µL/min for 3 min; mobile phase A (0.1% formic acid) and mobile phase B (0.1% formic acid in acetonitrile). After desalting and pre-concentration, the peptides were eluted from the precolumn to the analytical column and separated with a gradient of 3–40% mobile phase B over 90 min at a flow rate of 300 nL/min, followed by 10 min at 90% B. The column temperature was maintained at 35°C. Mass accuracy was maintained by use of a lock mass ([Glu^1^]-Fibrinopeptide B) that was delivered at 250 nL/min at a concentration of 100 fmol/µL to the reference spray.

Mass spectrometric analysis of tryptic peptides was performed using a Q-TOF Premier mass spectrometer (Waters Corporation). For all measurements, the mass spectrometer was operated in V-mode with a typical resolution of at least 10,000 FWHM. The TOF analyzer of the mass spectrometer was externally calibrated with a NaI mixture from m/z 50 to 1990. The data were post-acquisition lock mass corrected using the doubly charged monoisotopic ion of [Glu^1^]-fibrinopeptide B (m/z 785.8426). The reference spray was sampled at 60 s intervals. Accurate mass LC-MS data were collected in an alternating, low energy, and elevated-energy mode of acquisition (LC-MS^E^). The spectral acquisition time in each mode was 1.5 s with a 0.1 s interscan delay. In low energy MS mode, data were collected at constant collision energy of 4 eV. In elevated-energy MS mode, the collision energy was ramped from 15 to 40 eV during each 1.5 s integration. One cycle of low and elevated-energy data was acquired every 3.2 s. LC-MS data were processed and searched using ProteinLynx Global Server (PLGS) software, version 2.4.

Protein identifications were obtained using the PLGS search engine, using an IPI *Gallus gallus* database. Identification required three fragment ion matches per peptide, seven fragment ion matches per protein and a minimum of one peptide match per protein. The digestion reagent was trypsin, one missed cleavage was permitted, the fixed modification was carbamidomethylation at cysteine residues and the variable modification was oxidation of methionine residues. For label-free quantification, yeast alcohol dehydrogenase (ADH, accession P00300, 50 fmol injected on column) was used as a standard. The three most intense peptides were then used for quantification relative to the ADH standard [42]. Each sample was run in triplicate, and the three triplicate LC MSE analyses were treated as independent data sets for hierarchical cluster analysis; in all instances, the triplicate data sets were clustered most closely, as would be expected. The individual analyses are retained in the presentation of the data. A decoy version of the database is generated ‘on the fly’ with every search conducted to infer peptide and protein level false positive identification rates. The allowed protein FDR was initially set at 4%, which typically accumulates to a peptide FDR<1%. However, none of the initial decoy identifications passed these filtering criteria; hence, the protein FDR and subsequently the peptide FDR of identification in the reported list of identifications are both close to zero.

### Quantification of equalized proteins using QconCAT

Starting material and beads containing equalized protein were diluted 1∶10 with ammonium bicarbonate (50 mM), to which [^13^C_6_]arg, [^13^C_6_]lys-labelled QconCAT protein [35] was added (150 pmol to the starting material, 36 pmol to bead preparations containing equalized proteins). Protein was digested with trypsin at a ratio of protein: enzyme of 20∶1 with incubation at 37°C for 24 h. To ensure complete digestion, digested protein was analyzed by 1D SDS-PAGE to confirm the absence of intact proteins.

### LC-MS

For preliminary quantification, peptide mixtures were analyzed by LC-ESI-QTOF MS using an EASY-nLC (Proxeon, Odense, Denmark) nanoflow system coupled to a QTOF micro (Waters Corporation, Manchester, UK). Nanoflow HPLC at 200 nL/min was used to resolve peptides (in 0.1% v/v formic acid) over a 60 min acetonitrile gradient (0–100%). Peptides were acquired over the mass range 400–2000 m/z with the capillary voltage set at 1900 V, collision energy 10 V and sample cone at 55 V for the entire 60 min gradient. Q-peptides from proteins quantified as significantly enriched by the equalization process were confirmed by MS/MS (collision energy 30 V) and *de- novo* sequencing. For later studies, peptides were analyzed by LC-ESI-LTQ MS/MS using an Ultimate 3000 HPLC system (Dionex, Camberley, UK) coupled to a LTQ (Thermo Finnigan, Astmoor, UK). Nanoflow HPLC at 300 nL/min was used to resolve peptides (in 0.1% formic acid) over a 60 min acetonitrile gradient (0–100%). Peptides were acquired over the mass range 400–1500 m/z with the capillary voltage set at 50 V, spray voltage at 1.8 kV. Extracted ion chromatograms for heavy labeled Q-peptides were used for comparison of MS signal intensity of analyte and standard peptides.

### LC-MS/MS

For protein identification, 1D gel separations of starting material and beads containing equalized proteins were divided into 22 slices, each of which was de-stained using 50∶50 acetonitrile:50 mM ammonium bicarbonate, dehydrated with acetonitrile and digested overnight in-gel with trypsin. Resulting peptide solutions were analyzed by LC-ESI-LTQ MS/MS as described previously. MS/MS spectra were converted to dta files using BioWorks™ browser rev.3.3.1 (Thermo; MW range 400–3500, absolute threshold 10, precursor ion tolerance 2.5AMU, group scan 10, count 1, minimum ion count 1) and a Mascot generic format file was generated for all dta files. Data were searched against all Uniprot entries for *Gallus gallus* (database prepared on 30^th^ April 2009, 10973 entries) using MASCOT (in house) from which only confident identifications (MOWSE score >34, p<0.05) were accepted. Search parameters consisted of trypsin as the proteolytic enzyme, one missed cleavage, no fixed modifications, oxidation of methionine as a variable modification, mass tolerance of the precursor ion set at 250 ppm with a tolerance of 0.6 Da for the fragment ions.

## Results and Discussion

**Figure 1:**
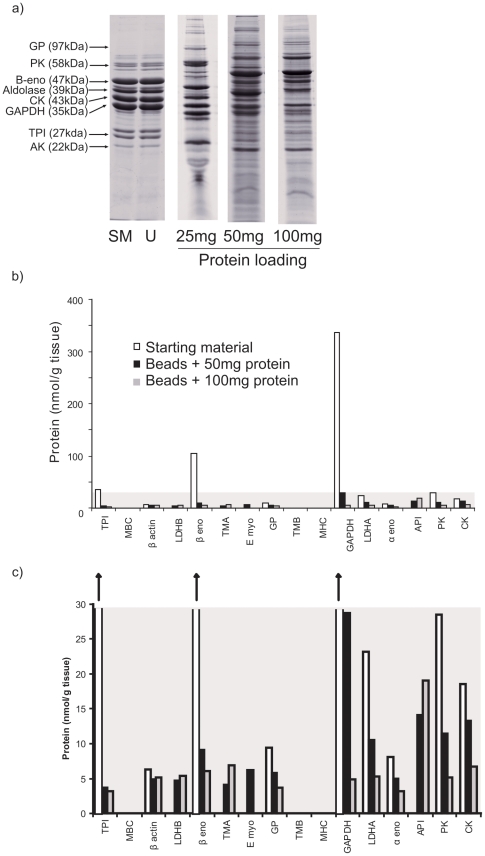
Equalization of skeletal muscle proteins by Prospectrum II equalization. a) Prospectrum II beads (20 mg) were exposed to increasing amounts of soluble protein from chick skeletal muscle (0–100 mg protein loading) in a final volume of 1.0 mL of 20 mM sodium phosphate buffer, pH 7.0. After 2 h, the beads were washed exhaustively (8 successive washes in the same buffer). The beads were recovered, suspended in SDS-PAGE sample buffer and applied directly to a 12.5% (w/v) acrylamide gel along with starting material (SM) and unbound protein (U; from 50 mg protein loading) washed from the beads, prior to staining with Coomassie blue. Individual slices from the gels were analyzed by LC-MS/MS (Table S1). b) To a 10-fold dilution of the bead suspension (diluents 50 mM ammonium bicarbonate) a QconCAT (36 pmol) designed for quantification of chicken skeletal muscle proteins was added, and the entire suspension plus QconCAT was digested to completion with trypsin (protein∶ protease ratio, 20∶1). Resultant peptides were analyzed by LC-MS and the bound proteins were quantified by extraction of the relevant ion chromatograms for the analyte or QconCAT standard peptides. Data are expressed relative to the protein abundance in the original tissue sample and data are included for the un-equalized starting material. c) The lowest panel is an expanded magnification of the shaded area in the b), to emphasize the equalization of lower abundance proteins. Abbreviations: TPI: triosephosphate isomerase; LDHB: lactate dehydrogenase isoform B; β-eno: beta enolase; TMA: tropomyosin A; GP: glycogen phosphorylase; TMB: tropomyosin B; MHC: myosin heavy chain; GAPDH: glyceraldehyde-3-phospate dehydrogenase; LDHA: lactate dehydrogenase isoform A, α-eno: alpha enolase, API: actin polymerization inhibitor, PK: pyruvate kinase; CK: creatine kinase.

The soluble fraction of mature (approximately 30 d after hatch) chicken skeletal muscle comprises relatively few, high abundance proteins that are pronounced on 1-D SDS-PAGE but confirmed by 2-D GE. These are typically glycolytic enzymes [30], [32], [33], [35], [36] that dominate many proteome analyses, but which are particularly evident in muscle that has a high proportion of fast twitch ‘white’ fibres, such as the chicken pectoralis muscle (Figure 1a). The wash fractions were analyzed in the same way to confirm that all unbound protein had been washed away from the beads (results not shown). The pattern of proteins on the gel changed considerably as proteins were loaded onto the beads. The largest differences were between the protein composition of the starting material and the bead contents at the lowest level of loading (25 mg). Several of the strongest bands on the gel in the starting material were absent from the loaded beads and new protein bands had appeared. As the loading doubled or quadrupled to 50 mg or 100 mg, the banding pattern continued to change, although the differences between the 50 mg and 100 mg loading were less pronounced. However, several major proteins were still evident on SDS-PAGE. To assess the change in protein composition of bead-bound proteins, 1D gels were sliced into segments and each slide was analyzed by LC-MS/MS (Figure S1, Table S1).

The extent of equalization was assessed by absolute quantification using stable isotope labeled internal standards. We have designed a QconCAT for the quantification of 20 high abundance soluble proteins in chicken skeletal muscle [35], [43] to absolutely quantify the change in protein expression of these proteins during growth from 1–30 d. QconCAT was added to starting material or beads coated with equalized protein, prior to digestion with trypsin. When using surrogate peptides for absolute quantification it is vital that complete digestion is achieved and the digested protein samples were analyzed by 1D SDS-PAGE after digestion to ensure the complete removal of intact proteins (results not shown). For absolute quantification, peptides were analyzed by LC-ESI-MS using relative signal intensity of analyte (light) and internal standard (heavy) peaks. The identity of the peptides used for absolute quantification was confirmed by MS/MS and *de- novo* sequencing (results not shown). This was expressed as nmol/g tissue before and after equalization, which after equalization lacks biological meaning but demonstrates in absolute terms the degree of protein equalization (Figure 1b,c) and permits comparison with previously published data. The extent to which the most abundant proteins, for example glyceraldehyde 3-phosphate dehydrogenase and β enolase have been diluted during equalization is striking and other proteins, previously undetectable, were enriched (for example, tropomyosin A and actin polymerization inhibitor). This is exactly the behavior expected through use of the equalization beads.

To obtain a more comprehensive assessment of equalization, a fixed quantity of Prospectrum beads (20 mg dry weight) was incubated with soluble proteins at a total load of 25 mg, 50 mg, 100 mg, 250 mg, 500 mg and 1000 mg total soluble protein and each was incubated for 2 h. The beads were washed to remove unbound protein and starting material, beads and unbound protein were analyzed by label-free proteomics, using data independent MS^E^ LC-MS acquisition, and combining the intensity of the top three precursor ions, giving protein identification data coupled with label-free quantification [37], [41], [42]. MS^E^ based analysis is a data-independent approach that makes use of alternating low energy and high energy scans, conducted at high repetition rates. The data from the high-energy scan therefore contain fragment ions from all peptides present in the chromatographic flow. Post-acquisition, the precursor∶product relationships are resolved by coincidence of retention time and by resolution of combinatorial possibilities of precursor∶product relationships [39], [40]. Moreover, the intensities of the three most intense peptide ions can be summed and yield, when compared to an internal standard digest (in these experiments, yeast alcohol dehydrogenase, accession P00330, 50 fmol applied to column), a reliable measure of the absolute abundance of each protein detectable in the mixture. Each loading was compared with the starting material, and was analyzed qualitatively (total proteins identified) and quantitatively (fmol each protein on column). Analyses were conducted in triplicate.

**Figure 2:**
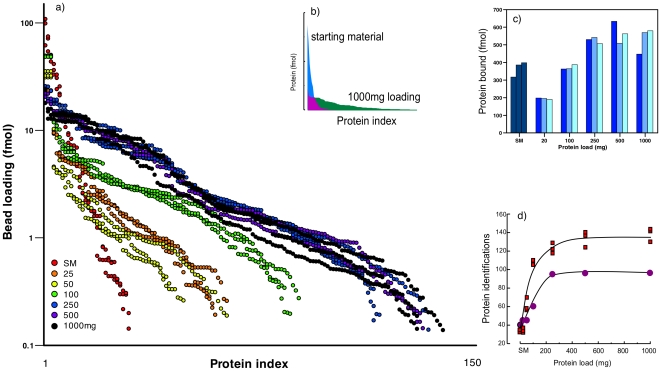
Gain in protein identification achieved by equalization. Prospectrum II beads were exposed to very high levels (up to 1000 mg protein/20 mg beads) of chick skeletal muscle soluble proteins in a final volume of 1.0 mL of 20 mM sodium phosphate buffer, pH 7.0. After 2 h, the beads were washed exhaustively (8 successive washes in the same buffer) and the suspension of beads was incubated with trypsin for digestion of bound proteins. The resultant peptides were analyzed by LC-MS/MS by data-dependent gel-LC-MS/MS and data independent LC-MS/MS. Panel a) shows the individually sorted abundance profiles as the beads were loaded from 25 to 1000 mg of starting protein (SM = starting material) and data are expressed as fmol on column. Each independent LCMS run was sorted independently from high to low abundance. The equalization was evident from the altered profile of the top 100 proteins on a linear abundance scale (panel b). The total protein bound is presented in panel c (n = 3) and number of proteins identified by label-free quantification (squares, n = 3) or gel-LCMS/MS (circles, n = 1) is given in panel d.

The un-equalised soluble protein fraction was first analysed by label-free proteomics using data independent data acquisition (MS^E^) (Figure 2a). For the starting material of soluble muscle protein, a total protein load of ∼350 pmol protein yielded a very limited set of positive identifications, consistent with the marked bias in protein content in this tissue and extending over 2.5 orders of magnitude in dynamic range. As a fixed quantity of beads was exposed to increasing amounts of protein, the distribution of protein abundances became more shallow, and extended over a higher number of bound proteins. The equalization is evident from the distribution of the top 100 most abundant proteins in starting material (actually, only 35 proteins) and the highest bead loading – the markedly shallow profile is highly evident when the data are plotted on a linear scale (Figure 2b). This is concordant with the observation of increasing quantities of bead-bound protein, from 200 fmol on column at 20 mg protein load to over 500 fmol on column at the highest protein loads (Figure 2c). In the starting material, about 35 proteins could be confidently identified, a common experience in crude analyses of total skeletal muscle soluble protein. As the protein load increased, so the number of identifications followed suit, but reached a plateau at about 140 proteins from 250 mg loading to 10000 mg loading (Figure 2d; this is mirrored by the total protein calculated on column for the three replicate analyses of increased protein loading, panel c). The identified proteins are a little higher in number than the equivalent numbers obtained by LC-MS/MS analysis of individual gel slices but the limitations of both sets of identifications is consistent with restricted complexity and dynamic range in each sample.

**Figure 3:**
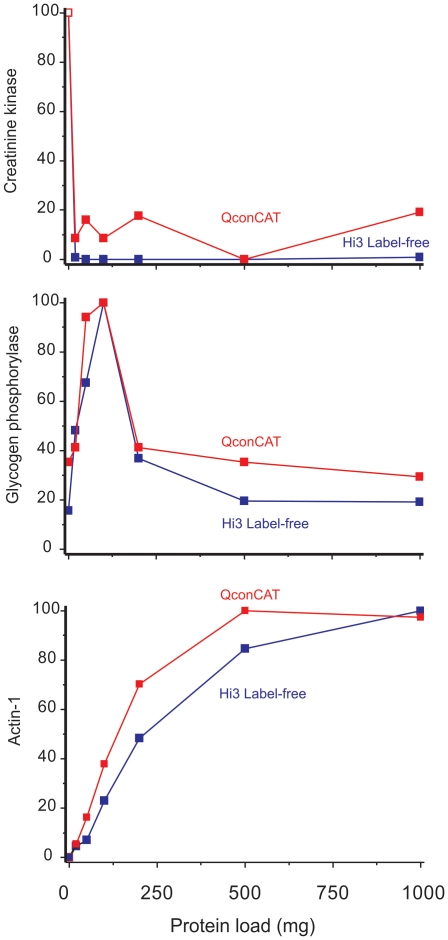
Comparison of individual protein behavior by QconCAT and label-free quantification. Prospectrum II beads were exposed to very high levels (up to 1000 mg protein/20 mg beads) and bound proteins were analyzed by quantitative proteomics, using both a label free approach in addition to a labelled internal standard (QconCAT). For three representative proteins, the binding behavior (reduction in representation: creatine kinase, transient binding: glycogen phosphorylase b and gradual accumulation: actin-1) was assessed by both quantitative approaches (QconCAT: open squares, label free: filled squares). To aid comparison, data are presented as a percentage of the highest amount of bound protein obtained in each analysis.

The proteins that were identified changed throughout the loading range, consistent with the changes observed in the 1D SDS-PAGE patterns. A total of 360 proteins were identified using LC-MS^E^ and 210 using GeLC-MS/MS (Table S2). To ensure the veracity of the label-free analysis in defining protein responses, profiles from three proteins demonstrating different loading behaviors were analyzed by QconCAT and label-free quantification (Figure 3). The behavior of the three proteins was almost identical, irrespective of the quantification method used, lending credence to the comparative analyses. Creatine kinase, present at high levels in the starting material, was bound at very low levels to the beads; glycogen phosphorylase exhibited a biphasic response, initially binding at high levels but then at lower levels when the protein load was increased. Finally, actin continued to bind at very high levels as the beads were loaded with more and more input protein. The behavior of the last two proteins was unexpected, as there was no reason *a priori* why a protein should bind to fewer beads when the protein load was enhanced. Moreover, the failure of actin to saturate was also unexpected. Both of these behaviours implied that protein∶protein interactions were taking place on the beads.

**Figure 4:**
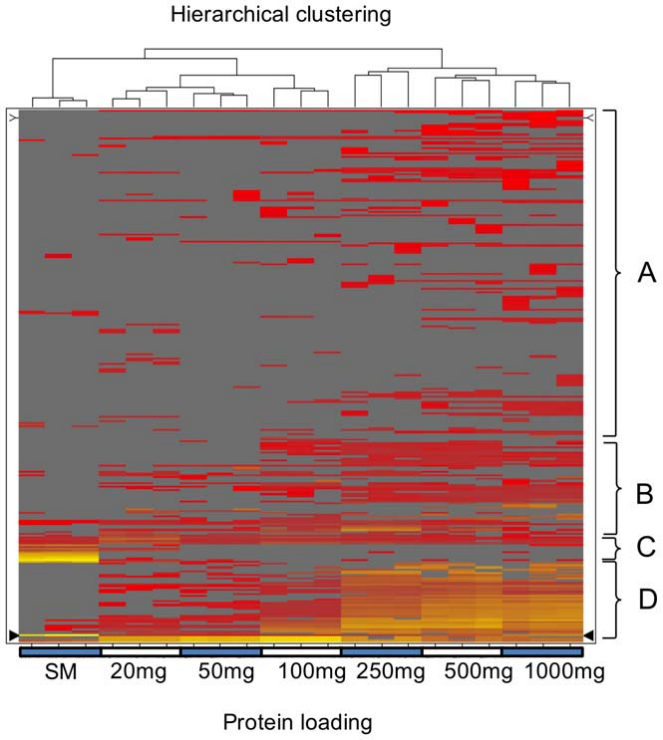
Global analysis of protein equalization. Prospectrum II beads were exposed to very high levels (up to 1000 mg protein/20 mg beads) and bound proteins were analyzed by quantitative label-free LC-MS. All quantitative data were expressed as heat diagrams (from red, low abundance to yellow, high abundance; grey: protein absent), arranged according to hierarchical (unweighted average) clustering of samples and K-means (data centroid based search initialized) clustering of the individual protein behaviors. Both clustering methods utilized Euclidian distance methods to assess similarity. The readily discernible classes of behaviors (labeled A ..D) are discussed in the text.

The comparison of QconCAT and label-free analysis validated the quantitative information from label-free analysis and the full data set for these proteins permitted a global exploration of the protein equalization of skeletal muscle proteins. When the entire data set, defining the abundance profiles for over 350 proteins and over seven loading levels was analyzed, hierarchical analysis sorted the different protein loadings according to the increasing mass (and concentration) of protein applied to the beads, and at all loading levels, correctly clustered the triplicate analyses together. In terms of the behavior of individual proteins, profile grouping by K-means clustering revealed four overall types of behavior. First, a small group of proteins (group C, Figure 4) were typified by very high concentrations in the starting material, but declined rapidly to practically undetectable levels as the beads were challenged with increasing quantities of muscle protein. These proteins, glyceraldehyde 3 phosphate dehydrogenase, enolase B, pyruvate kinase, lactate dehydrogenase A, aldolase A, phosphoglycerate kinase, creatine kinase, phosphoglucomutase, phosphoglycerate mutase, triose phosphate isomerase and adenylate kinase are the same proteins that are highly abundant in the 1D SDS-PAGE gel of the soluble proteins of skeletal muscle (Figure 1). Typically, the quantities of these proteins bound to the beads declines to about 1% of that in the starting material. This is the behavior that would be expected for combinatorial equalization, since the few beads capable of binding each of these proteins would rapidly become saturated when exposed to such high concentrations of cognate proteins. Two further classes (A and B, Figure 4) demonstrated broadly the same behavior – the progressive appearance in analysis at heavy loading of protein onto the beads. Group B generally accumulated more readily than group A. Again, this would be consistent with the expected behavior of the beads.

**Figure 5:**
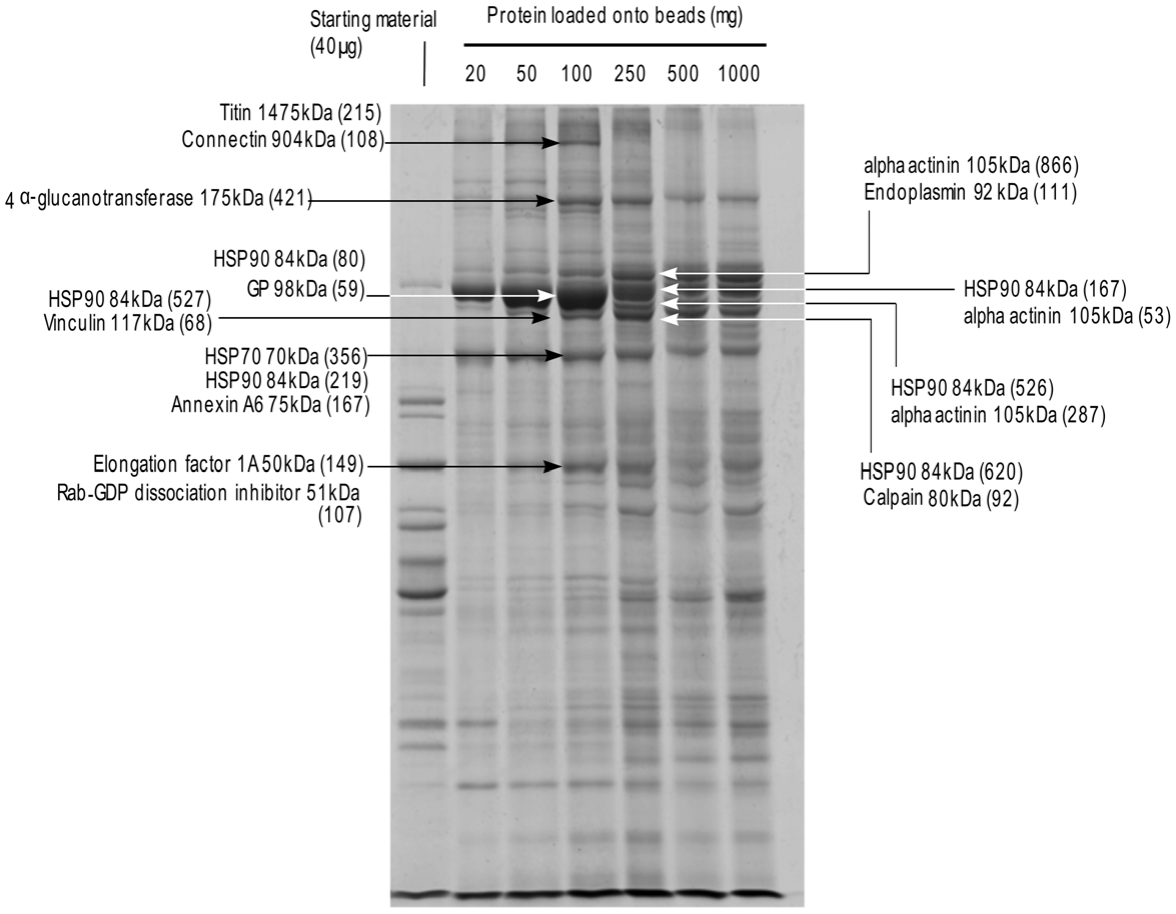
Equalization achieved by extreme bead loading. Prospectrum II beads were exposed to very high levels (up to 1000 mg protein/20 mg beads) of chick skeletal muscle soluble proteins in a final volume of 1.0 mL of 20 mM sodium phosphate buffer, pH 7.0. After 2 h, the beads were washed exhaustively (8 successive washes in the same buffer) and samples were analyzed by 1D SDS-PAGE as described in the legend to Figure 1. Bands were excised, digested with trypsin and analyzed by LC-MS/MS and selected identified proteins are highlighted on the gel.

However, a final class of proteins (Group D, Figure 4) was notable for unexpected and atypical behavior. This group of proteins was virtually undetectable in the starting material but accumulated on the beads to levels far higher than would be anticipated from the behavior of the majority of the other proteins. The proteins that exhibited this over-accumulation included heat shock proteins hsp90 and hsp70, α-actinin-2, calpains, initiation factors (E1α-1) and calmodulins, proteins that were also identified in 1D SDS-PAGE analysis of specific bands (Figure 5). Contrary to expectations, the SDS-PAGE gel did not show a uniform ‘smear’ of a complex, approximately equimolar, protein mixture. There was strong evidence for selective binding and accumulation of specific proteins, as evidenced by intensely staining bands on the gel even at the highest loadings. These bands were excised and identified by LC-MS/MS, and their identities are indicated on the gel. There was thus good consistency between the label-free quantification and the developing asymmetry on gel-based analyses.

The dynamics of bead loading should be expected to be complex. As a limited set of binding sites are specific for each protein, loading would reflect binding of each bead with a mixture of proteins, the composition of which would be dictated by *k*_on_, *k*_off_ and the solution concentration of the protein in the pool. At equilibrium loading, it would be anticipated that each site was fully saturated with specific proteins, resulting in a near-equimolar representation of the proteins present in the starting material. The intensity of protein bands in the pattern seen at 100 mg loading may reflect the saturation of beads with specific proteins that were present in adequate concentrations – as such, the density of these bands might reflect the intensity seen at saturation. If the beads were exposed to additional starting material, each protein reaching saturation might achieve the same intensity – true equalization. A proteome sample should then become progressively more equalized as the beads are exposed to greater and greater amounts of a protein mixture. Although low loadings of the beads had achieved some degree of equalization, we explored whether the degree of equalization would improve if a large excess of starting material was loaded on to the beads. In principle, more and more individual proteins should saturate the beads at approximately the same capacity. Notable proteins that were present in the lanes at higher loading included heat shock proteins, actinin and some very high molecular weight structural muscle proteins such as titin and connectin. None of these proteins were evident from LC-MS/MS analyses of the starting material, and therefore had been concentrated considerably by the beads.

Two explanations are offered for this behavior. First, the hexapeptide library on the bead might contain sufficient hydrophobic peptides to permit binding of proteins such as heat shock proteins to multiple beads, leading to asymmetric equalization. However, this is not a particularly satisfactory explanation for the other proteins that demonstrate similar behavior. The second explanation is that the beads can act as scaffolds for the assembly of complex networks that can accumulate in successive layers on the bead surface. The heat shock proteins are able to bind a wide range of proteins with exposed hydrophobic surfaces, and their marked predominance in the heavily loaded beads suggests that this is the case. The marked enhancement of HSP90 and α-actinin imply the development of a large assembly of protein complexes consisting primarily of these proteins, implying a preferential association between these molecules.

**Figure 6:**
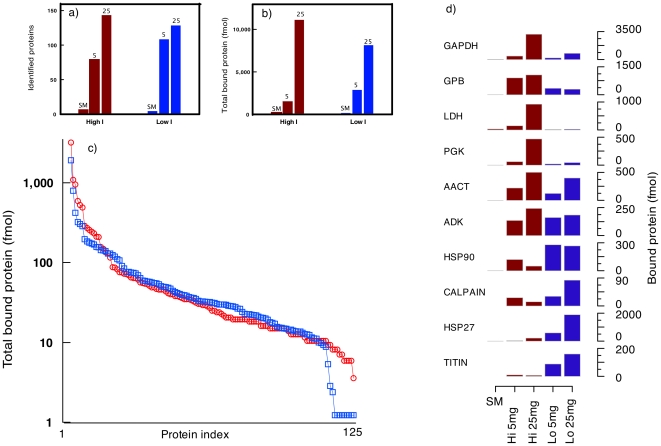
The effect of ionic strength on asymmetric protein loading. Prospectrum II beads were loaded with protein at two different levels (5 mg and 25 mg) in buffers at two different ionic strength values (Low, 20 mM sodium phosphate buffer, pH 7.5, I = 50 mM; High, 20 mM sodium phosphate buffer, pH 7.5 containing 150 mM NaCl, I = 200 mM). After loading, the proteins bound to the beads under each condition and the total bead loading (in fmol protein) were analysed by label-free quantification. The number of proteins identified are presented in panel a) and the total on-column yield of protein is given in panel b). The entire protein profile (irrespective of the identity of the proteins) is presented in panel c) and the behavior of specific proteins was highlighted for both sets of loadings and ionic strength values (panel d). Key: GAPDH: glyceraldehyde-3-phosphate dehydrogenase; GPB: glycogen phosphorylase b; LDH: lactate dehydrogenase; PGK: phosphoglycerate kinase; AACT: alpha actinin; ADK: adenylate kinase; HSP90: heat shock protein 90; HSP27: heat shock protein 27.

The interaction between the hexapeptides in the library and the target proteins should be expected to vary from protein to protein. Electrostatic interactions would be anticipated to diminish at high ionic strength, whereas for salts such as NaCl used here, hydrophobic interactions should be strengthened. The pattern of protein binding could therefore be expected to depend on solution conditions. To test this, portions of the hexapeptide beads were incubated with skeletal muscle soluble proteins in two buffers, one at low ionic strength (50 mM) and one at high ionic strength (200 mM). Slightly more protein was bound to the beads at high ionic strength (Figure 6a), behavior that would be consistent with enhancement of hydrophobic interactions. At these low protein loadings, similar numbers of proteins were bound, with slightly more proteins being retained by the beads at the higher ionic strength. However, this overall description conceals the variation elicited by ionic strength. Of 222 proteins bound to the beads at 25 mg load, 107 were exclusively bound at high ionic strength, 41 proteins were bound at low ionic strength and 74 were bound at both ionic strength values. For the proteins bound at both solution conditions, the ratio of the quantity of protein bound at high∶low ionic strength values varied considerably, from over 30∶1 (lactate dehydrogenase A) to 0.03∶1 (titin). It is clear that the solution conditions can have a profound influence on the protein binding profile, which opens up new opportunities for extending the scope and range of the equalization profiles. To illustrate the difference in behavior of individual proteins at different ionic strength values, representative proteins are included in Figure 6. Some proteins, notably the glycolytic enzymes such as glyceraldehyde-3-phospate dehydrogenase or glycogen phosphorylase are bound to a greater extent in high ionic strength buffers. Others, such as the heat shock proteins and titin, bind much more extensively at low ionic strength values. Finally, proteins such as alpha actinin are bound strongly, irrespective of the solution conditions. Careful selection of solution conditions might therefore not only diminish protein assemblies but also be used to extend the range of proteins that are accessible. This might usefully be extended in an orthogonal complementarity to adjustment of pH, as has recently been successfully demonstrated [27], [45].

Most studies using bead-based proteome equalization have focused on biological fluids, and in particular plasma although recently, these equalization analyses have been extended to some rather more exotic proteomes [46]–[48]. Although it has a high dynamic range of protein expression, plasma does not contain high concentrations of heat shock proteins, and, almost by virtue of their function in plasma, true plasma proteins might not be anticipated to have exposed hydrophobic surfaces, although serum albumin is known to have exposed hydrophobic regions. However, any tissue sample would be anticipated to be replete with heat shock proteins, and the behavior that is observed here with the soluble proteins of skeletal muscle might be replicated with other tissue samples. For cellular material or tissues, the quest for the ‘democratic proteome’ [18] may well be thwarted by the inability of some proteins to behave as anticipated, democratically, and indeed, subvert the behavior of other proteins. If the hypothesis of a heat shock protein framework turns out to be proven in other tissues, then equalization of tissue proteomes may remain elusive, unless bead-loading conditions can be elucidated to minimize or eliminate the effect.

## Supporting Information

Figure S1 **Protein identification following equalization of chicken skeletal muscle proteins.** For protein identification, 1D gel separations of starting material and beads containing equalized proteins were divided into 22 slices, each of which was de-stained and digested overnight in-gel with trypsin. Resulting peptide solutions were analyzed by LC-ESI-LTQ MSMS and MSMS data were searched against all Uniprot entries for *Gallus gallus* (database prepared on 30^th^ April 2009, 10973 entries) using MASCOT from which only confident identifications (MOWSE score>50, p<0.05) were accepted, for details see Table S1.
(PDF)

Table S1 **Protein identifications derived from in-gel analyses.**
(XLSX)

Table S2 **Protein identification and label-free quantification of bead-bound proteins.**
(XLSX)
