# Supplementary figures and images for: Asymmetric Proteome Equalization of the Skeletal Muscle Proteome Using a Combinatorial Hexapeptide Library
Source: PLoS One. 2011 Dec 19;6(12):e28902. doi: 10.1371/journal.pone.0028902 (PMC3242751; doi:10.1371/journal.pone.0028902)

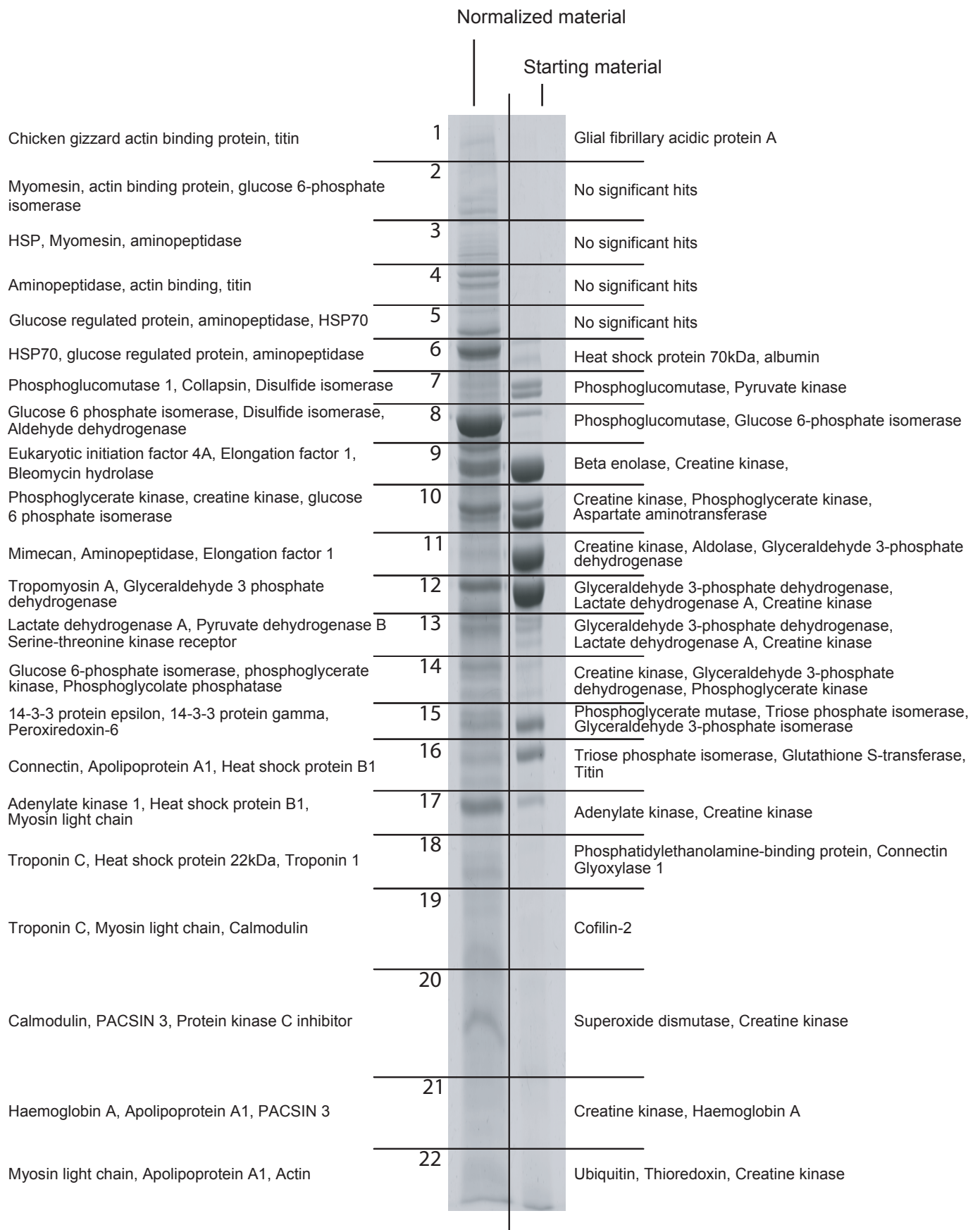

Supplement: Figure S1 — Protein identification following equalization of chicken skeletal muscle proteins. For protein identification, 1D gel separations of starting material and beads containing equalized proteins were divided into 22 slices, each of which was de-stained and digested overnight in-gel with trypsin. Resulting peptide solutions were analyzed by LC-ESI-LTQ MSMS and MSMS data were searched against all Uniprot entries for Gallus gallus (database prepared on 30th April 2009, 10973 entries) using MASCOT from which only confident identifications (MOWSE score>50, p<0.05) were accepted, for details see Table S1. (PDF) [file pone.0028902.s001.pdf]
